# P-1889. Integrating One Health into Medical Education: A Student-Led, Practice-Based Interdisciplinary Approach to Building Collaborative Health Preparedness

**DOI:** 10.1093/ofid/ofaf695.2058

**Published:** 2026-01-11

**Authors:** Allison W Cheung, Mary Cormier, David Grace, Elizabeth A Scruggs-Wodkowski, Laura Power

**Affiliations:** University of Michigan Medical School, Ann Arbor, Michigan; University of Michigan Medical School, Ann Arbor, Michigan; University of Michigan School for Environment and Sustainability, Ann Arbor, Michigan; Veteran Affairs Ann Arbor Healthcare System; University of Michigan Medical School, Ann Arbor, Michigan; University of Michigan School of Public Health, Ann Arbor, Michigan

## Abstract

**Background:**

As human health threats evolve, medical education must keep pace. One Health offers a critical framework for addressing challenges like climate change, pandemics, and antimicrobial resistance, yet remains largely absent from medical curricula. A 2020 survey found only 56% of medical schools included One Health content. To address this gap, we created the One Health Student Consortium (OHSC), a student-driven initiative at the University of Michigan (UM) designed to integrate One Health principles into medical education through interdisciplinary, practice-based collaboration. Here we describe OHSC’s design and early impact.

**Figure:**
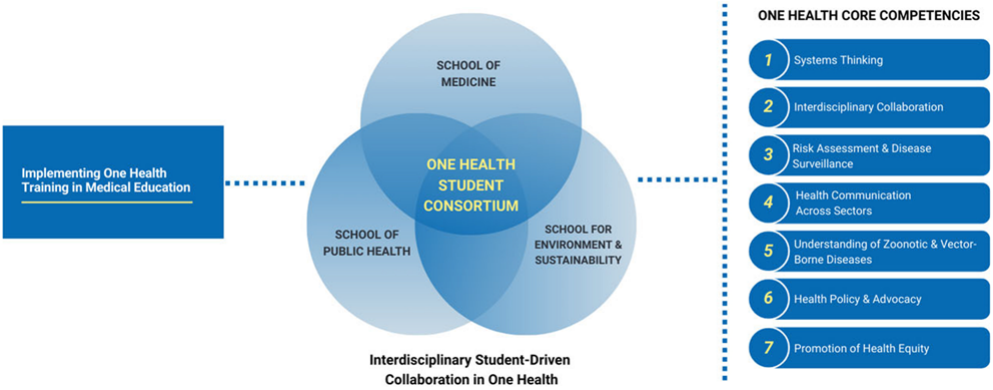
Schematic for the integration of One Health core competencies into medical training

**Methods:**

Students from the UM Schools of Medicine, Public Health, and Environment launched OHSC in 2023 to create interdisciplinary One Health opportunities. Educational initiatives included speaker events on zoonotic disease emergence, environmental toxicology, and low-cost One Health clinics. Students also engaged in practice-based activities, including developing a policy brief proposing evidence-based strategies for avian influenza control in Michigan, guided by health policy experts. To support professional development, OHSC created a travel stipend for conferences and designed a One Health certificate to formally recognize sustained interdisciplinary engagement in One Health.

**Results:**

Since its launch, OHSC has engaged over 180 students across seven UM schools, including 32% medical, 50% public health, and 15% environmental students. A pilot survey of 31 participants in 2024 revealed that 89% were interested in more One Health coursework, cross-enrollment, guest lectures, and access to research opportunities. OHSC has now secured institutional funding, faculty mentorship, and partnerships with Public Health Prepared and Center for Global Health Equity, establishing a strong foundation for growth.

**Conclusion:**

OHSC’s interdisciplinary support and collaboration from its inception suggests an ongoing need for increased One Health opportunities in medical education. Future directions include creating a cross-campus research database and expanding community partnerships, positioning OHSC as a potential model for other institutions seeking to strengthen One Health training.

**Disclosures:**

All Authors: No reported disclosures

